# The Inhibitory Effects of Maclurin on Fatty Acid Synthase and Adipocyte Differentiation

**DOI:** 10.3390/ijms25168579

**Published:** 2024-08-06

**Authors:** Ji Young Hwang, Hyeon Hak Jeong, Jiwon Baek, Jiyun Lee, Heeyeon Ryu, Jae-Il Kim, Bonggi Lee

**Affiliations:** 1 Department of Smart Green Technology Engineering, Pukyong National University, Busan 48513, Republic of Korea; hjy0506@pukyong.ac.kr (J.Y.H.); wjdgusgkr123@nate.com (H.H.J.); 2 Department of Food Science and Nutrition, Pukyong National University, Busan 48513, Republic of Korea; loog_ood@naver.com (J.B.); g0320g@gmail.com (J.L.); heeyeon3115@naver.com (H.R.); jikim@pknu.ac.kr (J.-I.K.); 3 Marine Integrated Biomedical Technology Center, The National Key Research Institutes, Pukyong National University, Busan 48513, Republic of Korea

**Keywords:** maclurin, fatty acid synthase, adipogenesis, obesity, 3T3-L1 adipocytes

## Abstract

Obesity is a complex health condition characterized by excessive adipose tissue accumulation, leading to significant metabolic disturbances such as insulin resistance and cardiovascular diseases. Fatty acid synthase (FAS), a key enzyme in lipogenesis, has been identified as a potential therapeutic target for obesity due to its role in adipocyte differentiation and lipid accumulation. This study employed a multidisciplinary approach involving in silico and in vitro analyses to investigate the anti-adipogenic properties of maclurin, a natural phenolic compound derived from *Morus alba*. Using SwissDock software (ChEMBL version 23), we predicted protein interactions and demonstrated a high probability (95.6%) of maclurin targeting FAS, surpassing the interaction rates of established inhibitors like cerulenin. Docking simulations revealed maclurin’s superior binding affinity to FAS, with a binding score of −7.3 kcal/mol compared to −6.7 kcal/mol for cerulenin. Subsequent in vitro assays confirmed these findings, with maclurin effectively inhibiting FAS activity in a concentration-dependent manner in 3T3-L1 adipocytes, without compromising cell viability. Furthermore, maclurin treatment resulted in significant reductions in lipid accumulation and the downregulated expression of critical adipogenic genes such as PPARγ, C/EBPα, and FAS, indicating the suppression of adipocyte differentiation. Maclurin shows potential as a novel FAS inhibitor with significant anti-adipogenic effects, offering a promising therapeutic avenue for the treatment and prevention of obesity.

## 1. Introduction

Obesity is mainly associated with excessive energy intake, resulting in the accumulation of white adipose tissue (WAT). In individuals with obesity, the expansion of the adipose depot, particularly hypertrophy, characterized by an increase in the size of existing adipocytes, plays a major role in metabolic syndrome and more so than hyperplasia, the formation of new adipocytes [1].

Hypertrophied adipocytes secrete adipokines, potentially disrupting insulin signaling and attracting macrophages. The increased presence of macrophages induces an increase in pro-inflammatory cytokines and a decrease in the net flux of fatty acids into the adipocyte [2,3]. Consequently, fatty acids accumulate more in the form of triacylglycerides in non-adipose tissues, including the liver and skeletal muscle. Metabolic disorders, such as type 2 diabetes mellitus, or cardiovascular disease, are closely linked to the dysregulation of adipogenesis and fatty acid metabolism in obesity [3]. There are several factors involved in regulating adipogenesis, including peroxisome proliferator-activated receptors (PPARs), the CCAAT-enhancer binding protein family (C/EBP), the sterol regulatory element-binding protein family (SREBP), and fatty acid synthase (FAS). PPARγ, which is the master regulator of adipogenesis, initiates adipogenesis by leading to fat cells having various functions of mature adipocytes [4,5]. C/EBPα is crucial for terminal adipogenesis, not only to control insulin action, but also to sustain the expression of PPARγ in mature adipocytes [5].

Understanding the adipocyte formation process induced by transcription factors provides valuable insights into the regulatory mechanisms of adipocyte development and function, contributing to the discovery of therapies for preventing obesity [6]. Additionally, FAS plays a pivotal role in inducing the differentiation of preadipocytes into mature adipocytes [7]. Fatty acids serve as crucial precursors for cell survival as they not only contribute to the formation of phospholipids, essential components of membranes, but also serve as energy sources. However, excessive FAS activity and fatty acids prompt cells to initiate various reactions to prevent overaccumulation [8]. These responses include stimulating β-oxidation for degradation, synthesizing triglycerides for storage in lipid droplets, and inhibiting endogenous fatty acid synthesis. Consequently, the surplus accumulation of fatty acids can lead to lipotoxicity, thereby contributing to the development of metabolic syndrome [8]. Given the central role of FAS in mature adipocyte formation and the link between excessive fatty acids and lipotoxicity, inhibiting FAS may be crucial for suppressing excessive adipogenesis and regulating fatty acid metabolism. Therefore, while well-known FAS inhibitors such as C75 have demonstrated efficacy in inhibiting FAS and regulating fatty acid metabolism [9], there remains a need for the further investigation of effective FAS inhibitors to explore potential improvements in managing obesity.

Maclurin, a natural phenolic compound predominantly in *Morus alba* (white mulberry), has shown efficacy as a melanogenesis inhibitor, suppressing tyrosinase activation and melanin accumulation in UVB-induced B16F10 cells [10], and exhibited anticancer effects on human non-small-cell lung cancer cells, and human osteosarcoma cells [11,12]. Maclurin treatment inhibited aryl hydrocarbon receptor signaling and activated antioxidant response element signaling, indicating its potential to protect against benzo[a]pyrene pollutants via Nrf2-mediated pathways [13]. Furthermore, while previous research suggests its potential anti-obesity effects, such as reducing lipid accumulation and triacylglycerol contents during 3T3-L1 adipocyte differentiation with mango peel and seed extracts containing maclurin [14], its effects on fatty acid synthesis specifically remain unexplored. Given that maclurin is a xanthone precursor molecule, and previous studies have demonstrated that xanthones found in *Garcinia mangostana* possess properties that combat fat formation by decreasing the expression of PPARγ and the activity of FAS [15], further investigation into maclurin’s potential as an FAS inhibitor is warranted. Therefore, our study aims to provide novel insights into the anti-adipogenic properties of maclurin by regulating FAS activity. In this study, we specifically focus on maclurin’s capacity to inhibit fatty acid synthesis through in vitro and in silico analyses. This research may contribute to the comprehensive understanding of its anti-adipogenic effects and suggest its potential utility in obesity management.

## 2. Results

### 2.1. Prediction of Maclurin Targeting FAS

**Figure 1:**
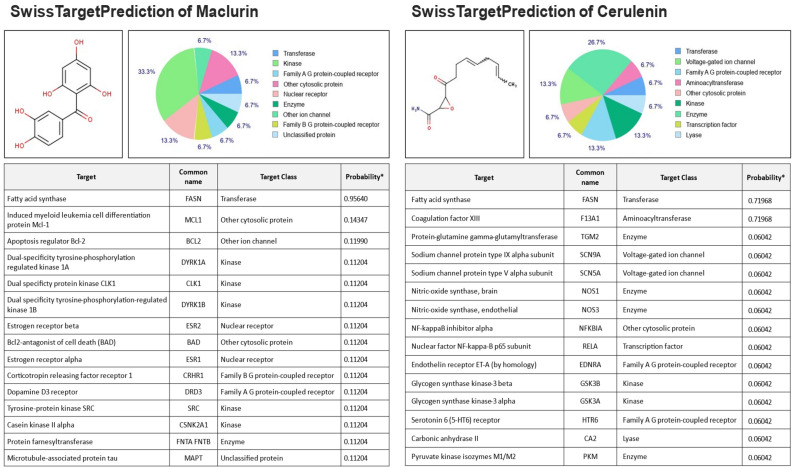
The SwissTargetPrediction report files were obtained using cerulenin and maclurin as query molecules, determining potential target proteins. The predicted binding affinity for maclurin with the fatty acid synthesis enzyme was 95.6%, while cerulenin showed a value of 71.9%. Probability*: The probability predicted by SwissTargetPrediction, accounting for the statistical significance of the prediction model.

In our study, we employed the SwissDock software to predict potential protein interactions of maclurin (Figure 1). The analysis revealed diverse possibilities of interactions with various proteins. While the probabilities of interaction with induced myeloid leukemia cell differentiation protein Mcl-1, apoptosis regulator Bcl-2, and dual-specificity tyrosine phosphorylation-regulated kinase 1A were each below 15%, the probability of targeting FAS was notably high at 95.6%. FAS is an essential enzyme for synthesizing fatty acids, and its regulation can significantly impact cellular fatty acid synthesis capacity. Therefore, the high probability of interaction between maclurin and FAS suggests that maclurin may modulate the activity of this enzyme, thereby influencing cellular fatty acid synthesis. Moreover, compared to the well-established FAS inhibitor cerulenin, which exhibited a 75% interaction rate, maclurin demonstrates a higher potential for interaction with FAS.

### 2.2. Docking Simulation between Maclurin and FAS

**Figure 2:**
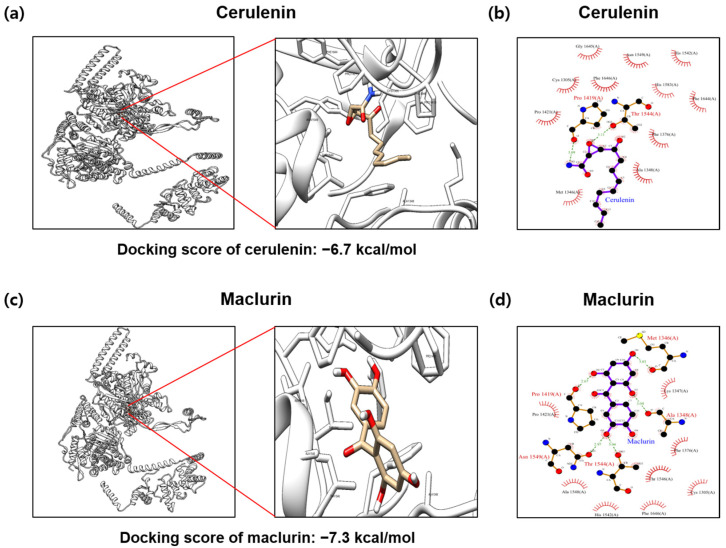
In silico docking simulation of fatty acid synthesis enzyme protein and maclurin. Auto−Dock Vina 1.1.2 software was utilized to predict the ligand-binding capability of maclurin, indicating strong inhibitory activity against the fatty acid synthesis enzyme protein. The binding affinity between the fatty acid synthesis enzyme protein (PDB ID: 2VKZ) and (**a**) cerulenin (CID: 5282054), and (**c**) maclurin (CID: 68213) was evaluated. In a marked square box, it represents the fatty acid synthesis enzyme protein binding site with an enlarged image. The analysis of binding residues for (**b**) cerulenin, (**d**) maclurin, and the FAS protein was expressed in ligplot.

Protein–ligand docking simulations were conducted to predict the binding affinity of maclurin and cerulenin with FAS. Our analysis focused on comparing the docking scores to assess the binding strength of maclurin at the known binding site for the FAS inhibitor cerulenin. The predicted binding affinity between FAS and cerulenin is −6.7 kcal/mol (Figure 2a), whereas maclurin exhibits a binding affinity of −7.3 kcal/mol (Figure 2c), signifying a greater affinity compared to cerulenin. The analysis of interactions between the amino acid residues of FAS protein and its ligands utilized the PDBsum database. According to structural predictions, cerulenin forms two hydrogen bonds (Pro 1419, Thr 1544) and involves 11 non-ligand residues in hydrophobic contacts (Gly 1645, Asn 1549, His 1542, Cys 1305, Phe 1646, His 1583, Phe 1644, Pro 1421, Phe 1376, Ala 1348, Met 1346) (Figure 2b). Maclurin forms five hydrogen bonds (Ala 1348, Pro 1419, Met 1346, Thr 1544, Asn 1549) and involves eight non-ligand residues in hydrophobic contacts (Pro 1421, Lys 1347, Phe 1376, Thr 1546, Ala 1548, His 1542, Phe 1646, Cys 1305), suggesting a potential contribution to binding with FAS (Figure 2d).

### 2.3. Inhibition of FAS Enzyme Activity by Maclurin

**Figure 3:**
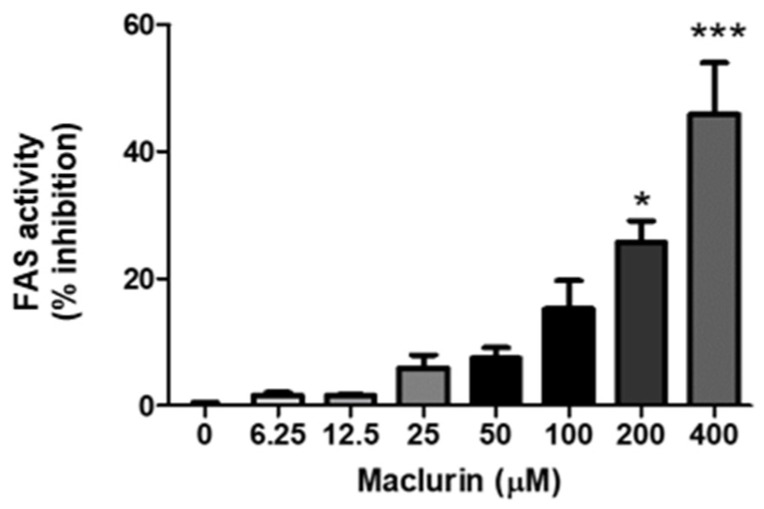
Illustrates the inhibitory effects of maclurin on fatty acid synthesis, as determined by the FAS activity assay (*n* = 3–4/group). The results are presented as mean ± SEM. * *p* < 0.05 and *** *p* < 0.001 indicate a significant difference compared to the untreated control group.

Due to the potential inhibitory activity of maclurin against FAS, as suggested by in silico analysis, we extracted crude enzyme fractions from fully differentiated adipocytes, and FAS-inhibitory effects were tested using FAS-related substrates. As depicted in Figure 3, when FAS was treated with various concentrations of maclurin (0–400 μM), we observed a concentration-dependent decrease in activity, confirming its potential for inhibiting FAS.

### 2.4. Effect of Maclurin on Cell Survival and Intracellular FAS Activity in 3T3-L1 Adipocytes

**Figure 4:**
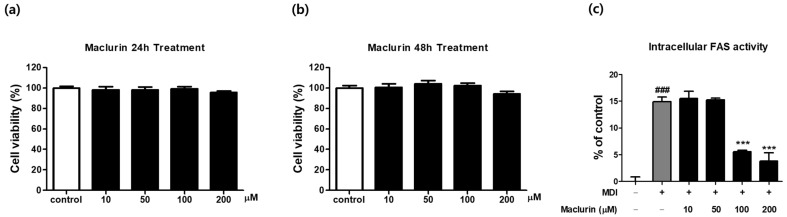
Cell viability was measured in 3T3-L1 preadipocytes treated with various concentrations (10–200 μM) of maclurin for (**a**) 24 h (*n* = 4–8/group) and (**b**) 48 h (*n* = 4–8/group). The effect of maclurin on FAS inhibition was assessed by measuring intracellular FAS activity (*n* = 3–4/group) in differentiated 3T3-L1 preadipocytes treated with (**c**) various concentrations of maclurin (10–200 μM). Results are presented as mean ± SEM. Significance levels are indicated as ### *p* < 0.001 compared to the untreated control group, and *** *p* < 0.001 compared to the MDI-treated group.

To investigate the effects of maclurin on cell viability and FAS activity, experiments were conducted using 3T3-L1 adipocytes. 3T3-L1 cells, widely utilized in studies related to adipocyte differentiation, serve as an ideal model for investigating fatty acid synthesis, as they faithfully replicate adipocyte characteristics. The viability of 3T3-L1 cells treated with various concentrations of maclurin (10–200 μM) was evaluated using MTS analysis (Figure 4a,b). Subsequently, intracellular enzyme activity analysis was performed to quantify FAS activity following maclurin treatment. FAS primarily catalyzes the synthesis of saturated fatty acid palmitate from acetyl-CoA and malonyl-CoA, utilizing NADPH as a reducing agent, in the endogenous lipogenesis pathway [16,17,18,19]. Our experimental results indicate that maclurin treatment did not affect cell viability within our tested concentrations. However, a significant decrease in FAS activity was observed in the 100 μM and 200 μM maclurin treatment groups (Figure 4c). These findings underscore the effectiveness of maclurin in inhibiting FAS activity within adipocytes.

### 2.5. Reduction in Fat Accumulation in 3T3-L1 Cells by Maclurin

**Figure 5:**
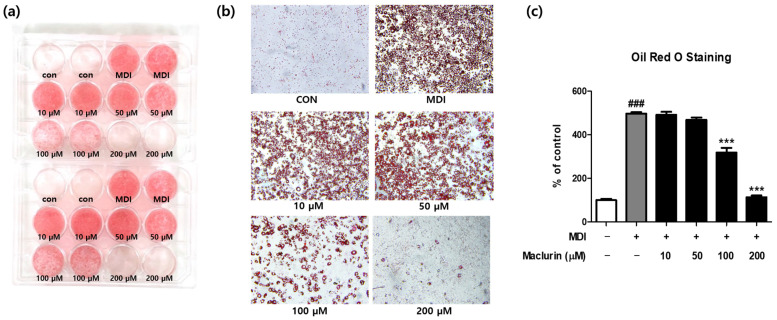
3T3-L1 adipocytes were treated with various concentrations of maclurin (10–200 μM) and harvested 8 days after the differentiation initiation for Oil Red O staining (**a**,**b**). The quantification of lipid accumulation was based on the optical density of (**c**) Oil Red O extracted from adipocytes at 500 nm (*n* = 4/group). Results are presented as mean ± SEM. Significance levels are indicated as ### *p* < 0.001 compared to the untreated control group, and *** *p* < 0.001 compared to the MDI-treated group. Scale bar: 100 μm.

In this study, we investigated the correlation between alterations in fatty acid synthesis and the morphological changes in adipocytes induced by FAS inhibition. Previous experiments confirmed maclurin’s effective inhibition of FAS, thereby impacting fatty acid synthesis. Subsequently, we assessed the effects of FAS inhibition on adipocyte differentiation and lipid accumulation. Since obesity onset is closely associated with lipid synthesis and accumulation [20], we employed Oil Red O staining to examine maclurin’s inhibitory effects on lipid accumulation. The data exhibited lipid droplet formation and accumulation with MDI treatment, whereas in the maclurin-treated groups, this process was dose-dependently reduced (Figure 5a). Additionally, microscopic observation confirmed that maclurin treatment decreased lipid accumulation (Figure 5b,c).

### 2.6. The Influence of Maclurin on Genes Involved in 3T3-L1 Adipocyte Differentiation

**Figure 6:**
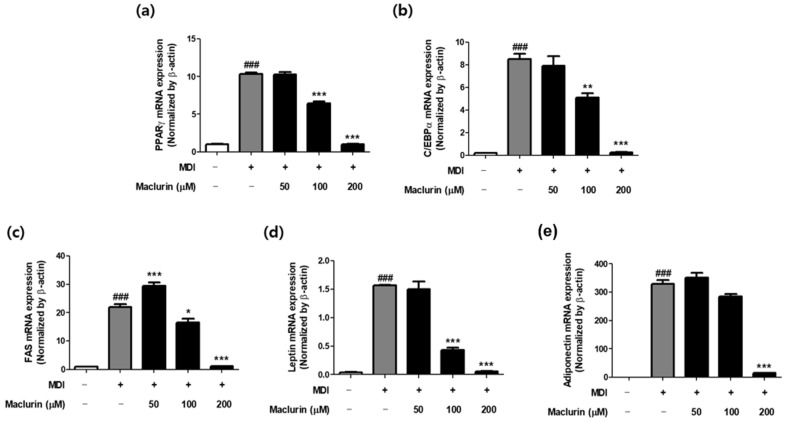
3T3-L1 adipocytes were treated with maclurin (50–200 μM) and harvested 8 days after differentiation initiation to determine the mRNA expression levels of (**a**) PPARγ, (**b**) C/EBPα, (**c**) FAS, (**d**) leptin, and (**e**) adiponectin. Results are presented as mean ± SEM. Significance levels are indicated as ### *p* < 0.001 compared to the untreated control group, and * *p* < 0.05, ** *p* < 0.01, *** *p* < 0.001 compared to the MDI-treated group.

PPARγ, C/EBPα, and C/EBPβ, key genes associated with lipid synthesis, play crucial roles in the early and intermediate stages of adipocyte differentiation. The differentiation process of 3T3-L1 preadipocytes into adipocytes is divided into early differentiation regulated by C/EBPβ and C/EBPδ, members of the CCAAT/enhancer-binding protein family, and late differentiation regulated by peroxisome PPARγ and C/EBPα. Stimulation by hormones leads to the increased expression of C/EBPβ and C/EBPδ, which in turn induces low levels of PPARγ and C/EBPα, facilitating the cooperative induction of each other’s gene expression [21]. MDI treatment significantly increased the mRNA levels of PPARγ, C/EBPα, FAS, leptin, and adiponectin, while maclurin treatment significantly reduced the mRNA levels of these genes (Figure 6 a–e). PPARγ is essential for adipogenesis, and cells lacking PPARγ exhibit significantly reduced levels of C/EBPα and decreased adipogenic capacity [22,23], thereby the sequential decrease in key markers like PPARγ reflects the inhibition of adipocyte differentiation. Therefore, the reduction in these markers signifies the inhibition of adipocyte differentiation induced by maclurin treatment. Moreover, this marker reduction suggests that maclurin effectively suppresses adipogenesis.

## 3. Discussion

The results of our study provide evidence supporting the potential of maclurin as an FAS inhibitor, exerting anti-adipogenic effects in 3T3-L1 adipocytes. FAS emerges as a crucial player in adipocyte formation, as it catalyzes the synthesis of fatty acids essential for cell survival and function [7]. However, excessive FAS activity contributes to lipid overaccumulation and subsequent lipotoxicity, exacerbating metabolic complications [8]. Through in silico analysis, we identified a high probability of maclurin targeting FAS, suggesting its potential as an FAS inhibitor. Docking simulations further supported this notion, revealing maclurin’s superior binding affinity to FAS compared to the established FAS inhibitor cerulenin. Experimental validation confirmed maclurin’s ability to inhibit FAS activity in 3T3-L1 adipocytes, leading to a concentration-dependent decrease in fatty acid synthesis. Importantly, maclurin treatment did not compromise cell viability, underscoring its specificity towards FAS inhibition without detrimental effects on cellular integrity. Consistent with FAS inhibition, maclurin treatment resulted in a significant reduction in lipid accumulation in 3T3-L1 adipocytes, as evidenced by Oil Red O staining and microscopic observations. Furthermore, maclurin’s impact on key adipogenic genes further elucidated its mechanism of action. Treatment with maclurin downregulated the expression of PPARγ, C/EBPα, and other markers associated with adipocyte differentiation, indicating its ability to suppress adipogenesis. Notably, the sequential decrease in these markers reflects the inhibition of adipocyte differentiation induced by maclurin treatment.

Our findings show that maclurin treatment not only inhibits FAS activity but also downregulates key adipogenic transcription factors such as PPARγ. By attenuating adipocyte differentiation, maclurin intervenes at a crucial metabolic node, impeding the accumulation of triglycerides within adipocytes. Maclurin’s demonstrated efficacy in reducing lipid accumulation in adipocytes suggests its potential as a therapeutic agent for preventing and treating obesity. The observed concentration-dependent decrease in lipid droplet size and number underscores its ability to modulate adipocyte morphology, a hallmark of adipogenic inhibition. Moreover, maclurin’s natural origin as a phenolic compound derived from *Morus alba* (white mulberry) underscores its appeal as a botanical therapeutic agent. Natural products have long been explored for their bioactive properties and perceived safety profiles, making maclurin an attractive candidate for further development for therapeutic application.

Based on our data, while the reduction in adipocyte differentiation may be beneficial for decreasing adipose tissue mass, it could potentially be harmful to energy homeostasis and metabolic regulation. The lipids that would normally be stored in adipocytes might accumulate in other tissues, such as the liver, leading to ectopic fat deposition and associated metabolic complications. This phenomenon necessitates a comprehensive evaluation of maclurin’s overall impact on metabolic health. Maclurin has been shown to exert diverse biological effects beyond its anti-adipogenic properties, including anti-inflammatory [24], antioxidant [25], and mesenchymal stem cell protective effects [26]. To thoroughly assess the overall effects of maclurin, including its potential benefits and drawbacks, comprehensive animal studies are essential. Thus, further animal studies should evaluate not only the anti-adipogenic and metabolic impacts but also the broader physiological effects to determine the therapeutic potential and safety of maclurin in the context of obesity treatment.

Additionally, fatty acid synthesis in adipocytes is relatively low compared to dietary and hepatic sources. Indeed, adipocytes primarily store fatty acids delivered via very low-density lipoproteins (VLDL) [27] rather than synthesizing them de novo from glucose [28]. Nevertheless, previous studies have used adipose tissue-specific FAS deletion mice (achieved by crossing FAS^lox/lox^ mice with adiponectin-Cre transgenic mice, resulting in FASKOF) to investigate the effects of reduced FAS activity in adipose tissue on weight gain and fat accumulation [29]. FASKOF mice exhibited decreased FAS protein expression in both white adipose tissue and brown adipose tissue, with a reduction in FAS enzyme activity observed specifically in white adipose tissue [29]. On the other hand, these changes did not affect the liver [29]. Additionally, when comparing FASKOF mice and control mice fed a normal diet, there were no differences in body weight changes or glucose tolerance [29]. However, when fed a high-fat diet, FASKOF mice exhibited reduced body weight, WAT weight, and size compared to the control group [29]. Additionally, a recent study revealed that removing the FAS in adipocytes, thereby inhibiting de novo lipogenesis, induces browning in inguinal white adipose tissue (iWAT) [30]. FAS KO in adipocytes decreased palmitate levels and increased the expression of de novo lipogenesis enzymes ACLY and ACC, as well as the levels of acetyl-CoA and malonyl-CoA [30]. Various adipose-specific KO mouse models were created, including ACLY, ACC1, ACC2, MCD, and double KOs such as ACLY/FAS, ACC1/FAS, and ACC2/FAS, to investigate how these metabolic changes affect iWAT browning [30]. In adipocytes, the inhibition of acetyl-CoA and malonyl-CoA induced by ACLY KO or ACC1 KO did not prevent the browning of iWAT [30]. Conversely, increasing malonyl-CoA levels in MCD KO mice did not induce browning [30]. Notably, adipose ACC1 KO, which blocks malonyl-CoA and palmitate synthesis, induced a robust thermogenic response in iWAT similar to FAS KO [30]. However, blocking malonyl-CoA through ACC2 KO did not induce UCP1 expression [30]. This indicates that ACC1 and FAS strongly suppress adipocyte thermogenesis [30]. Body composition results compared to controls showed lower body fat percentage in FAS KO but not ACC1 KO mice [30]. Despite no significant differences in food intake, physical activity, body mass, and adipocyte size, the FAS KO mice exhibited a high energy expenditure rate consistent with the browning phenotype [30]. Therefore, inhibiting fatty acid synthesis by deleting FAS or ACC1 promotes thermogenesis in white adipocytes and prevents the typical whitening process in brown adipose tissue [30]. This inhibition has the potential to activate thermogenesis and promote metabolic improvement by inhibiting fatty acid synthesis in white adipocytes [30]. It will be particularly interesting to further study whether maclurin affects thermogenesis and energy expenditure in animal models of obesity.

Interestingly, FAS deletion in adipocytes activates PPARα signaling pathways that stimulate lipid catabolism in adipocytes [29]. This phenomenon can be explained by the fact that FAS inhibition reduces the production of lipid ligands essential for activating PPARγ [29]. These lipids, synthesized by FAS, generate alkyl ether lipids through the PexRAP pathway, acting as endogenous ligands for PPARγ. The disruption of FAS decreases these ether lipids, altering the coactivator milieu to favor PPARα-dependent gene expression. This shift in the balance of lipid ligands from PPARγ to PPARα highlights a significant change in the regulatory landscape of lipid metabolism, promoting catabolic processes in adipocytes. Understanding this shift provides valuable insight into the molecular mechanisms underlying the metabolic effects observed with FAS inhibition [29].

Given these findings, it is crucial to investigate whether maclurin also activates PPARα signaling pathways. Understanding maclurin’s potential role in this mechanism could provide deeper insights into its effects on lipid metabolism and adipocyte function, further elucidating its potential therapeutic benefits in obesity and related metabolic disorders. Future studies will focus on examining the impact of maclurin on PPARα activation to determine if it similarly influences lipid catabolism through this pathway.

Additionally, despite its promising attributes, several challenges and avenues for future research warrant consideration. Firstly, elucidating maclurin’s pharmacokinetic profile, including its bioavailability, metabolism, and tissue distribution, is essential for optimizing dosing regimens and predicting therapeutic outcomes. Additionally, comprehensive preclinical studies, including animal models of obesity, are needed to validate maclurin’s efficacy, safety, and long-term effects on metabolic parameters. Furthermore, exploring synergistic interactions with existing anti-obesity agents or lifestyle interventions may enhance maclurin’s therapeutic efficacy and broaden its clinical applicability. Combining maclurin with dietary modifications, exercise regimens, or other pharmacological agents targeting complementary pathways could yield additive or synergistic effects in combating obesity.

In conclusion, maclurin inhibited FAS activity and reduced the mRNA levels of key adipogenic transcription factors, thereby suppressing adipocyte differentiation. Further animal studies are warranted to elucidate its efficacy and safety profile for clinical applications in combating obesity and associated comorbidities.

## 4. Materials and Methods

### 4.1. Prediction of Protein Targets

SwissDock (ChEMBL version 23) is an open server provided since 2014, designed as a web tool to predict potential protein targets for small molecules. To predict protein targets interacting with the structure of maclurin, we utilized the Swiss Dock docking software (www.swisstargetprediction.ch, accessed on 20 December 2019) following established methods [31]. The structural information of maclurin (CID: 68213) and cerulenin (CID: 5282054) was obtained from PubChem. The target prediction was performed using the homo sapiens database.

### 4.2. Protein–Ligand Docking Simulation

The docking simulation was performed using the open-source software Auto−Dock Vina 1.1.2 (http://vina.scripps.edu, accessed on 1 July 2024). Predictions of binding affinity were made employing the 3D structure of the FAS protein (PDB ID: 2VKZ) complexed with cerulenin, a well-established inhibitor of FAS. After the removal of the native ligand, the binding site for cerulenin (CID: 5282054) was recalculated, and an evaluation was performed to ascertain the potential binding of maclurin (CID: 68213) to the same site. The structural information for cerulenin and maclurin was sourced from PubChem. Subsequently, interaction analysis between the FAS protein and the ligand was performed using LigPlot from the PDBsum (https://www.ebi.ac.uk/thornton-srv/databases/pdbsum/, accessed on 20 July 2024) database.

### 4.3. Cell Viability Experiment

Cell viability was evaluated using the MTS assay (CellTiter96^®^ AQueous One Solution Cell Proliferation Assay Kit, Promega, Madison, WI, USA). Briefly, 3T3-L1 cells were seeded in 96-well plates. Upon reaching confluence, cells were maintained in a fresh growth medium. Subsequently, the cells were cultured in a differentiation medium supplemented with various concentrations of maclurin (0–200 μM) for 2 days. Following incubation, 5 μL of MTS solution was added to each well, and the plates were incubated at 37 °C for 1 and 4 h to allow color formation. The optical density was subsequently assessed at 490 nm, employing a microplate reader. The obtained absorbance values were presented as a percentage relative to the mean absorbance value of the untreated control, representing cell viability in response to maclurin treatment. This calculation involved dividing the absorbance of cells treated with different concentrations of maclurin by that of the control group and multiplying the result by 100.

### 4.4. 3T3-L1 Adipocyte Differentiation

The 3T3-L1 cells procured from the American Type Culture Collection (ATCC), Rockville, MD, USA, were cultured in Dulbecco’s modified Eagle’s medium (DMEM, WELGENE, Gyeongsan, Republic of Korea) supplemented with 10% heat-inactivated calf serum (BCS, WELGENE, Gyeongsangbuk-do, Republic of Korea) and 1% penicillin-streptomycin (P/S, WELGENE, Gyeongsangbuk-do, Republic of Korea). This culture was maintained at 37 °C in a humidified atmosphere containing 5% carbon dioxide and 95% air. The differentiation process was initiated by exposure to a medium containing 0.5 mM IBMX, 1 µM dexamethasone, and 1 μg/mL insulin (MDI, Sigma-Aldrich, Merck KGaA, Darmstadt, Germany). Upon initiating differentiation, which commenced on the second day, the cells were transitioned to DMEM supplemented with 10% fetal bovine serum (FBS, WELGENE, Gyeongsangbuk-do, Republic of Korea), 1% P/S, and 1 μg/mL insulin to continue the induction process. The culture medium was refreshed every 2 days until day 8, by which point more than 90% of the cells had matured into adipocytes with visible lipid droplets. Subsequently, to monitor lipid accumulation, the culture medium was alternated every 2 days, starting from the onset of differentiation, with DMEM supplemented with maclurin (10% FBS, 1% P/S, and 1 μg/mL insulin).

### 4.5. FAS Activity Assay

The FAS inhibition test was evaluated using a slightly modified method [12,13]. Crude enzyme fractions isolated from fully differentiated 3T3-L1 cells were employed for the experiments, and the protein content in the supernatant was measured using BCA analysis. Specifically, cells were harvested with trypsin-EDTA after 8 days of differentiation in DMEM containing MDI. Subsequently, mechanical lysis was performed using 150 μL of 0.2 mM phosphate buffer (pH 7.0) containing 125 μg/mL of a protease inhibitor cocktail. Following freeze–thaw cycles, centrifugation was carried out at 1500× *g* for 10 min at 4 °C. The supernatant was extracted and utilized for the experiments. We referred to the supernatant as crude enzyme fractions because they encompass a variety of enzymes, not solely FAS. For more specific activities, appropriate substrates tailored for FAS were utilized. To assess the FAS inhibition capability of maclurin, various concentrations of maclurin (0–400 μM) were treated in 100 μL of assay buffer containing 0.1 M potassium phosphate, 1 mM DTT, 1 mM EDTA, and pH 7.0. This buffer contained 5 mM NADPH, 0.4 mM malonyl-CoA, 0.25 mM acetyl-CoA, and 1% DMSO. All reagents used in the buffer were purchased from Sigma-Aldrich (Merck KGaA, Darmstadt, Germany). DMSO served as the negative control group. The reaction rate was monitored by measuring the decrease in NADPH absorbance at 340 nm using a 96-well microplate reader. Intracellular FAS activity was supplemented with maclurin in the DMEM medium along with differentiation inducers such as MDI for up to 8 days. Subsequently, crude enzyme was extracted from the cells using the same method and utilized for experimentation. All experiments were conducted at 37 °C.

### 4.6. Oil Red Staining of 3T3-L1 Cells

Fat accumulation in 3T3-L1 cells was evaluated using Oil Red staining. Cells were divided into control, MDI, and different maclurin concentration groups (10, 50, 100, 200 μM). Oil Red staining was conducted upon full cell differentiation. Briefly, fully differentiated cells were fixed using 10% neutral buffered formalin (BIOSESANG, Yongin, Republic of Korea), followed by rinsing with 60% isopropyl alcohol (B&J, Clayton, NC, USA), and subsequently stained with a 60% Oil Red O working solution (Sigma, Saint Louis, MO, USA). After rinsing with distilled water, the cells were observed under a microscope and treated with 100% isopropyl alcohol for Oil Red O extraction. The resulting solution was then transferred to a 96-well plate and measured at 500 nm to assess optical density.

### 4.7. Real-Time PCR

Total RNA was extracted from cells on day 8 following induced differentiation using RiboEx (GeneAll, Seoul, Republic of Korea). cDNA synthesis was conducted using the Primer Script RT Reagent kit (SMART GENE, Daejeon, Republic of Korea) with 1 μg of total RNA. Quantitative real-time PCR was performed using the TOPrealTM SYBR Green qPCR PreMIX (Enzynomics, Daejeon, Republic of Korea) and the QuantStudioTM 1 Real-Time PCR System (Applied Biosystems, Foster City, CA, USA). The expression levels of target genes, normalized to β-actin, were determined using the 2^−ΔΔCT^ method. The primer sequences used are available in Table 1.

### 4.8. Statistical Analysis

Data were presented as mean ± standard error of the mean. One-way analysis of variance (ANOVA) was performed using GraphPad Prism 5.0 (GraphPad Software, La Jolla, CA, USA). The significance between groups was determined using Dunnett’s post hoc test.

## Figures and Tables

**Table 1:** Primer sequences of quantitative real-time PCR.

Gene	Forward (5′ → 3′)	Reverse (3′ → 5′)
Mouse		
PPARγ	CAC AAT GCC ATC AGG TTT GG	GCT GGT CGA TAT CAC TGG AGA TC
C/EBPα	GTC GAC AAG AAC AGC AAC GA	TCA CTG GTC AAC TCC AGC AC
FAS	GCT GTG CTT GCA GCT TAC TG	GTC CTC AGA CTT GTG GCA G
Leptin	GAC CGG GAA AGA GTG ACA GG	AGA GCA ATC TGA CAC CAG CC
Adiponectin	ACG ACA CCA AAA GGG CTC AG	CGT CAT CTT CGG CAT GAC TG

## Data Availability

Data are contained within the article.
